# Assessment of obesity stigma and discrimination among Spanish subjects with a wide weight range: the OBESTIGMA study

**DOI:** 10.3389/fpsyg.2023.1209245

**Published:** 2023-09-18

**Authors:** Enric Sánchez, Andreea Ciudin, Ana Sánchez, Sonsoles Gutiérrez-Medina, Nuria Valdés, Lilliam Flores, Amelia Marí-Sanchis, Fernando Goñi, Marta Sánchez, Joana Nicolau, Concepción Muñoz, Olaia Díaz-Trastoy, Guillem Cuatrecasas, Silvia Cañizares, Marta Comas, Carolina López-Cano, Albert Lecube

**Affiliations:** ^1^Obesity Unit, Endocrinology and Nutrition Department, University Hospital Arnau de Vilanova de Lleida, Obesity, Diabetes and Metabolism (ODIM) Research Group, IRBLleida, University of Lleida, Lleida, Spain; ^2^Obesity Unit, Endocrinology and Nutrition Department, Hospital Universitari Vall d’Hebrón, Vall d’Hebron Research Institute, Autonomous University of Barcelona, Barcelona, Spain; ^3^Centro de Investigación Biomédica en Red de Diabetes y Enfermedades Metabólicas Asociadas (CIBERDEM), Instituto de Salud Carlos III (ISCIII), Madrid, Spain; ^4^Obesity Unit, Endocrinology and Nutrition Department, Complejo Hospitalario Universitario de Ferrol, Ferrol, Spain; ^5^Obesity Unit, Endocrinology and Nutrition Department, Hospital Universitario Rey Juan Carlos, Madrid, Spain; ^6^Obesity Unit, Endocrinology and Nutrition Department, Hospital Universitario de Cabueñes, Gijón, Spain; ^7^Obesity Unit, Endocrinology and Nutrition Department, Hospital Clínic Barcelona, Barcelona, Spain; ^8^Obesity Unit, Endocrinology and Nutrition Department, Hospital Universitario de Navarra, Pamplona, Spain; ^9^Obesity Unit, Endocrinology and Nutrition Department, Hospital Universitario de Basurto, Bilbao, Spain; ^10^Obesity Unit, Endocrinology and Nutrition Department, Hospital Universitario Doctor Negrín, Las Palmas de Gran Canaria, Spain; ^11^Obesity Unit, Endocrinology and Nutrition Department, Hospital Universitario Son Llàtzer, Palma, Spain; ^12^Obesity Unit, Endocrinology and Nutrition Department, Hospital Universitario Reina Sofia de Córdoba, Córdoba, Spain; ^13^Obesity Unit, Endocrinology and Nutrition Department, Complejo Hospitalario Universitario de Pontevedra, Pontevedra, Spain; ^14^Obesity Unit, Endocrinology and Nutrition Department, Clínica Sagrada Familia-CPEN Barcelona, Health Science Department, UOC University, Barcelona, Spain; ^15^Obesity Unit, Psychiatry and Psycology Department, Hospital Clínic Barcelona, Barcelona, Spain; ^16^Clinical Psycology and Psycobiology Department, Universitat de Barcelona, Barcelona, Spain

**Keywords:** stigma, discrimination, weight bias, obesity, bariatric surgery

## Abstract

**Introduction:**

This study aims to assess the extent of rejection and instances of stigmatization linked to obesity within the Spanish population, encompassing a diverse spectrum of weights ranging from normal weight to morbid obesity. Additionally, the study seeks to identify the primary factors influencing these experiences and further examines the impact of bariatric surgery on such dynamics.

**Materials and methods:**

Multicenter observational study with involving a total of 1,018 participants who were recruited from various Obesity Units. Negatives attitudes towards people with obesity were assessed through three questionnaires: (i) Antifat Attitudes Scale (AFA), (ii) Stigmatizing Situations Inventory (SSI) and (iii) Weight Bias Internalization Scale (WBIS). Subjects were categorized into four groups based on their BMI and history of prior bariatric surgery.

**Results:**

The cumulative score across all questionnaires (AFA, SSI and WBIS) exhibited a progressive increase, from participants with normal weight to those with obesity (*p* < 0.001 for all). Within the AFA questionnaire, males showed more rejection towards people with obesity than women, also perceiving obesity as a disease linked to a lack of willpower (*p* = 0.004 and *p* = 0.030, respectively). The overall SSI score was negatively associated with age (*r* = −0.080, *p* = 0.011), with young participants encountering more stigmatizing experiences than their adult counterparts. Neither employment status nor educational demonstrated a significant association with any of the questionnaires. Interestingly, patients who underwent lost weight following bariatric surgery did not exhibit improved outcomes.

**Conclusion:**

Individuals with obesity demonstrate a heightened level of aversion towards the disease compared to those with normal weight. Concurrently, the incidence of stigmatizing encounters displays a concerning escalation among younger individuals.

## Introduction

Obesity is a chronic and relapsing disease that has acquired epidemic dimensions in the current era, due to its epidemiology that affects more than 20% of the world population (Hales et al., 2020). Obesity can be defined using the body mass index, which is a simple and widely utilized measure calculated by dividing a person’s weight in kilograms by the square of their height in meters. In the last 30 years, investigations have revealed that weight gain is significantly influenced by biopsychosocial factors, which often surpass individual decisions and accountability (Masood and Moorthy, 2023). So, multiple genes interacts with elements such as physical inactivity, excessive calorie consumption, conditions during prenatal development, post-birth influences, inadequate sleep, medication usage, medical conditions, socioeconomic standing, ethnic background, psychological stress, the impact of endocrine-disrupting chemicals, and the composition of the gastrointestinal microbiome to promote weight gain (Lin and Li, 2021; Masood and Moorthy, 2023). This chronic, relapsing, and multifactorial disease in its origin not only has a direct negative impact in the quality of life of patients, as it can affect various aspects of physical function, sexual life, self-steem, and social well-being, leading to a range of challenges and limitations (Caixàs et al., 2013). In addition, obesity also can shorten their life expectancy because it is the gateway to other diseases such as type 2 diabetes, cardiovascular disease, and some types of cancer (Di Angelantonio et al., 2016; Caballero, 2019). In the same way, obesity causes a great economic impact, both due to direct health costs and indirect costs secondary to decreased productivity, work absenteeism, and total disability (Destri et al., 2022).

There is increasing evidence linking obesity with higher prevalence of mood disorders such as anxiety and depression compared with the normal-weight population, especially in women in a society fueled by the cult of thinness (Daníelsdóttir et al., 2010; Sikorski et al., 2016; Spahlholz et al., 2016). But people living with obesity are also subject to discrimination, prejudice, and negative attitudes in society because of their weight (Vadiveloo and Mattei, 2017). Negative adjectives such as “clumsy,” “lazy,” “vulgar,” “rejected” and “lonely” have been largely used to define people with obesity (Teachman et al., 2003; Jáuregui Lobera et al., 2008). In this way, data from a large representative telephone survey in Germany demonstrated an overall prevalence of weight-based discrimination of 7.3%, which increased four-fold to 18.7% in participants with a body mass index between 35.0 and 39.9 kg/m^2^ (Spahlholz et al., 2016). Altogether, stigma in the context of obesity, can be described as the social and psychological process of discrediting and devaluing individuals due to their weight, leading to negative stereotypes and discrimination. This phenomenon can have profound effects on various aspects of an individual’s life, including mood, body image, and even job opportunities (Vadiveloo and Mattei, 2017). It is important to note that social discrimination and the feeling of rejection perceived by people with obesity have a potential effect on the lack of follow-up and adherence to dietary-behavioral treatment. In a cross-sectional study conducted on a sample of 5,129 people in the United States, weight discrimination was clearly associated with overeating, more frequent consumption of prepared foods, and less regular meal timing (Sutin et al., 2016). Interestingly, weight stigma may persist even substantive weight loss following bariatric surgery procedures, a set of surgical interventions designed to treat obesity and medical conditions related to excess weight (Dimitrov Ulian et al., 2023). This data suggest that the achievement of a thinner body is not always free to be associated with perceived negative judgment and condemnation by others.

As weight bias is an issue that is becoming increasingly important in the holistic approach to patients with obesity, reducing negative attitudes, beliefs, and stigmatization due to obesity would be a key factor in the battle against this growing public health concern. However, the information of the Spanish population on the discrimination and stigmatization suffered by people with obesity is still scarce. Hence, our aim was to address this gap by investigating the level of rejection and experiences of stigma related to obesity within the Spanish population, encompassing individuals with a diverse spectrum of weights, ranging from normal weight to morbid obesity. For this purpose, we administered three well-validated questionnaires as the Antifat Attitudes Scale (AAS), Stigmatizing Situations Inventory (SSI) and the Weight Bias Internalization Scale (WBIS) (Crandall, 1994; Myers and Rosen, 1999; Durso and Latner, 2008). Furthermore, we sought to identify the primary factors associated with these outcomes and examine the impact of bariatric surgery (BS).

## Methods

### Ethic statement

This study protocol was reviewed and approved by the ethics committee of the Arnau de Vilanova University Hospital, approval number CEIC-2190. In this study, the ethical guidelines of the Declaration of Helsinki and Spanish legislation on the protection of personal data have also been followed. Participants were asked to join voluntarily and were not awarded any financial or other compensation. Written informed consent was obtained from all subjects who agreed to take part in the study before completing the questionnaires.

### Study design and data collection

The OBESTIGMA study is a cross-sectional observational study in which we analyzed data from 1,126 participants collected between January 2019 and December 2021. Men and women aged 18 years or older and a body mass index (BMI) equal or greater than 18.5 kg/m^2^ were recruited from 13 Obesity Units in Spain. Patients who attended medical check-ups for their obesity, as well as their companions, were included. A total of 57 participants were excluded due to serious illness that determined a life expectancy of less than 6 months, intellectual disability and psychiatric illness not considered stable and/or failure to report weight and height. In addition, 51 patients refused to participate in the study (response rate of 95.4%). Consequently, the final sample consisted of 1,018 participants.

Weight and height data were obtained on the same day of the interview. Based on these data, the body mass index was calculated, and the respondents were classified into one of the following three groups: (i) subjects with healthy weight (from 18.5 to 24.9 kg/m^2^); (ii) overweight subjects (from 25.0 to 29.9 kg/m^2^); and (iii) subjects with obesity (≥30.0 kg/m^2^). Additionally, participants who reported having undergone bariatric surgery previously were categorized into a distinct fourth group. In addition to weight and height, the following information was recorded: age, sex, marital status, ethnicity, employment status, educational level, annual income, and self-perception of weight.

To ensure the independence, accuracy, and honesty of their responses, participants were given standardized verbal instructions. They were informed that there were no right or wrong answers, and that their responses would remain anonymous and confidential. Data collection took place in the outpatient waiting room. The majority of participants took 10–15 min to complete the survey.

### Assessment of negatives attitudes toward people with obesity

Three questionnaires were administered to all participants. The AFA is a tool developed by Crandall (1994) to measure prejudice against people with obesity in the United States. The scale is a numerical response scale, with a range between 1 (“not at all agree”) and 7 (“strongly agree”). It includes 13 questions divided into three subscales: dislike (rejection towards people with overweight or obesity), fear of being fat (fear of gaining weight) and willpower (weight is controllable). To obtain the score, the average of all the responses of each subscale and the global one were calculated. High scores on this scale are associated with strong attitudes against obesity. The validated version in Spanish of the AFA, with a good reliability results (Cronbach’s alphas of 0.78, 0.87, and 0.81 for dislike, fear of being fat and willpower respectively) was used (Molero et al., 2012; Macho et al., 2022).

The SSI is one of the most used methods to measure the weight stigma experiences, but it may be impractical due to its length (Myers and Rosen, 1999). Consequently, the brief version of the SSI is a more efficient tool for assessing experiences with weight-related stigma (Cronbach’s alphas in different samples ranging from 0.94 to 0.98) (Vartanian, 2015). This questionnaire assesses the stigmatizing experiences associated with being overweight that may have occurred to a subject at least once in his/her lifetime. The result is obtained by calculating the average of all responses. The higher the score, the greater the number of exposures to stigmatizing experiences.

The WBIS is a useful tool for assessing prejudice about overweight. This questionnaire identifies patients who need medical help to deal with weight stigma and had high internal consistency (Cronbach’s alphas = 0.90) (Durso and Latner, 2008). Composed of 11 items, it measures the degree to which the respondents believes that negative stereotypes about people with overweight and obesity apply to themselves. Responses range from “strongly disagree” to “strongly agree.” Items 1 and 9 should be scored reversely. The final score is obtained by calculating the average of all responses. High scores on the WBIS are related to strong anti-obesity attitudes.

### Statistical analysis

Statistical analyzes were performed using SSPS statistical package (IBM SPSS Statistics for Windows, Version 20.0. Armonk, NY, USA). The normal distribution of the variables was evaluated using the Shapiro–Wilk test. Given their normal distribution, quantitative data are expressed as the mean ± SD. Comparisons between groups were made using the ANOVA test for quantitative variables and Pearson’s chi-squared for categorical variables. The relationship between continuous variables was evaluated using the Pearson’s correlation test. Three multivariable logistic regression models (enter mode) were performed to evaluate the results of the questionnaires, including the following confounding factors in the analysis: age, sex, BMI, employment status, and educational level. Model calibration was assessed using the Chi-squared goodness-of-fit test. All “P” values were based on a two-sided test of statistical significance. The traditional significance level of *p* < 0.05 was chosen to find a middle ground between reducing false positive (Type I) errors and avoiding missing real effects (Type II) errors. The level of 0.05 was applied to all statistical analyses.

## Results

The detailed data for the 1,018 participants in the study, encompassing demographic, social, and economic characteristics, is presented in Table 1. It is observed that the progression through the BMI categories, from normal weight to obesity, was associated with increments in the age of participants and the percentage of women, married, unemployed, participants without a university education and housewife.

**Table 1:** Main clinical data of the study population according to their weight classification and those who underwent bariatric surgery.

	Normal weight (*n* = 173)	Overweight (*n* = 134)	Obesity (*n* = 572)	Bariatric surgery (*n* = 139)	*P*
BMI (kg/m^2^)	21.7 ± 1.9	27.2 ± 1.4	41.9 ± 7.4	33.5 ± 6.8	<0.001
Female, *n* (%)	135 (78.0)	89 (66.4)	398 (69.6)	108 (77.7)	0.029
Age (years)	39.7 ± 14.1	47.1 ± 13.5	48.3 ± 11.5	50.5 ± 10.0	<0.001
Caucasian, *n* (%)	127 (73.4)	91 (68.4)	385 (67.5)	87 (63.0)	0.692
Married, *n* (%)	106 (61.3)	97 (72.4)	372 (65.0)	103 (74.1)	0.018
Housewife, *n* (%)	6 (3.5)	9 (6.7)	125 (21.9)	35 (25.2)	<0.001
Unemployment, *n* (%)	14 (8.1)	14 (10.4)	125 (21.9)	35 (25.2)	<0.001
University studies, *n* (%)	120 (69.4)	58 (43.3)	134 (23.4)	25 (18.0)	<0.001
Income <20.000€, *n* (%)	87 (50.6)	77 (57.5)	348 (61.3)	99 (71.2)	0.065

Data are *n* (percentage) and mean and standard deviation; BMI, body mass index; €, euros.

The scores obtained in the three questionnaires (AFA, SSI, and WBIS) are shown in Table 2. In the AFA questionnaire, the total score progressively increased from participants with normal weight to participants with obesity. When the three subscales of the AFA were analyzed, different results were observed. Both the fear of being fat and willpower subscales showed a similar increase through BMI like the global score. However, antipathy towards obesity (dislike subscale) does not vary between the different groups. Finally, the results in the global score and in the three subscales were similar when patients who underwent bariatric surgery were considered.

**Table 2:** Scoring of the questionnaires according to the BMI of the study population.

	Normal weight (*n* = 173)	Overweight (*n* = 134)	Obesity (*n* = 572)	Bariatric surgery (*n* = 139)	*P*
AFA	33.7 ± 12.0	35.5 ± 11.0	38.6 ± 12.1	37.8 ± 12.5	<0.001
AFA_dislike	13.8 ± 6.4	13.1 ± 6.0	13.4 ± 7.3	13.6 ± 7.6	0.821
AFA_fear of being fat	8.7 ± 4.6	10.2 ± 4.9	13.8 ± 5.4	14.2 ± 5.4	<0.001
AFA_willpower	11.1 ± 4.3	12.1 ± 4.3	11.4 ± 4.9	10.2 ± 4.9	0.008
SSI	4.8 ± 9.6	11.8 ± 12.5	28.2 ± 17.7	27.7 ± 17.5	<0.001
WBIS	24.2 ± 11.5	30.2 ± 13.6	41.7 ± 15.2	40.9 ± 14.6	<0.001

Data are mean and standard deviation; AFA, Antifat Attitudes Scale; SSI, Stigmatizing Situations Inventory; WBIS, Weight Bias Internalization Scale.

**Figure 1:**
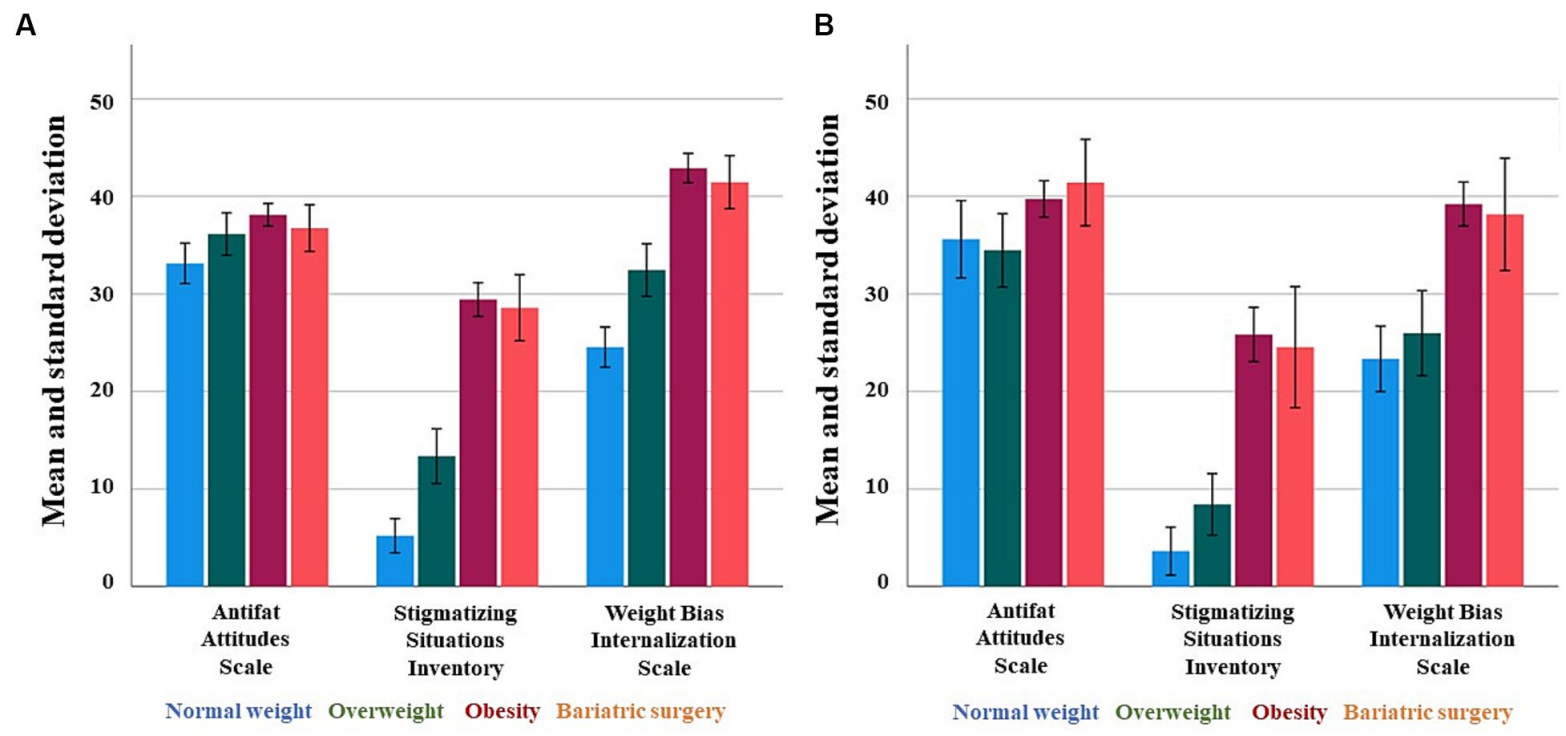
Results of the questionnaires according to four groups included in the study in female **(A)** and male **(B)** population.

When analyzing the results of the AFA questionnaire through a gender lens, significant disparities emerged once more. It was observed that men exhibited a higher inclination towards disapproving of individuals with overweight or obesity compared to women (*p* = 0.004) as depicted in Figure 1A. Moreover, men tended to associate obesity with a lack of willpower (*p* = 0.030). Conversely, women demonstrated a greater prevalence of fear concerning weight gain (*p* = 0.017) (Figure 1B).

On the SSI questionnaire, the number of exposures to stigmatizing experiences increased progressively with weight across the three groups. It is important to note that the overall SSI score was negatively associated with age, demonstrating that young participants experienced significantly more stigmatizing experiences than adults (*r* = −0.080, *p* = 0.011). In individuals with obesity, the three items from the SSI questionnaire that received higher scores were “Having people assume you overeat or binge eat because you are overweight” (negative assumption domain), “Not being able to find clothes that fit” (physical limitation donain), and “Being stared at in públic” (being stared at domain).

In the WBIS questionnaire, our observations revealed that individuals with obesity tend to more frequently attribute negative stereotypes to themselves. This inclination stems from a greater internalized bias toward obesity when compared to groups with lower weights. Finally, like the AFA questionnaire, the effect of bariatric surgery did not affect the perception of patients regarding stigmatizing situations and the internalization of weight bias.

**Figure 2:**
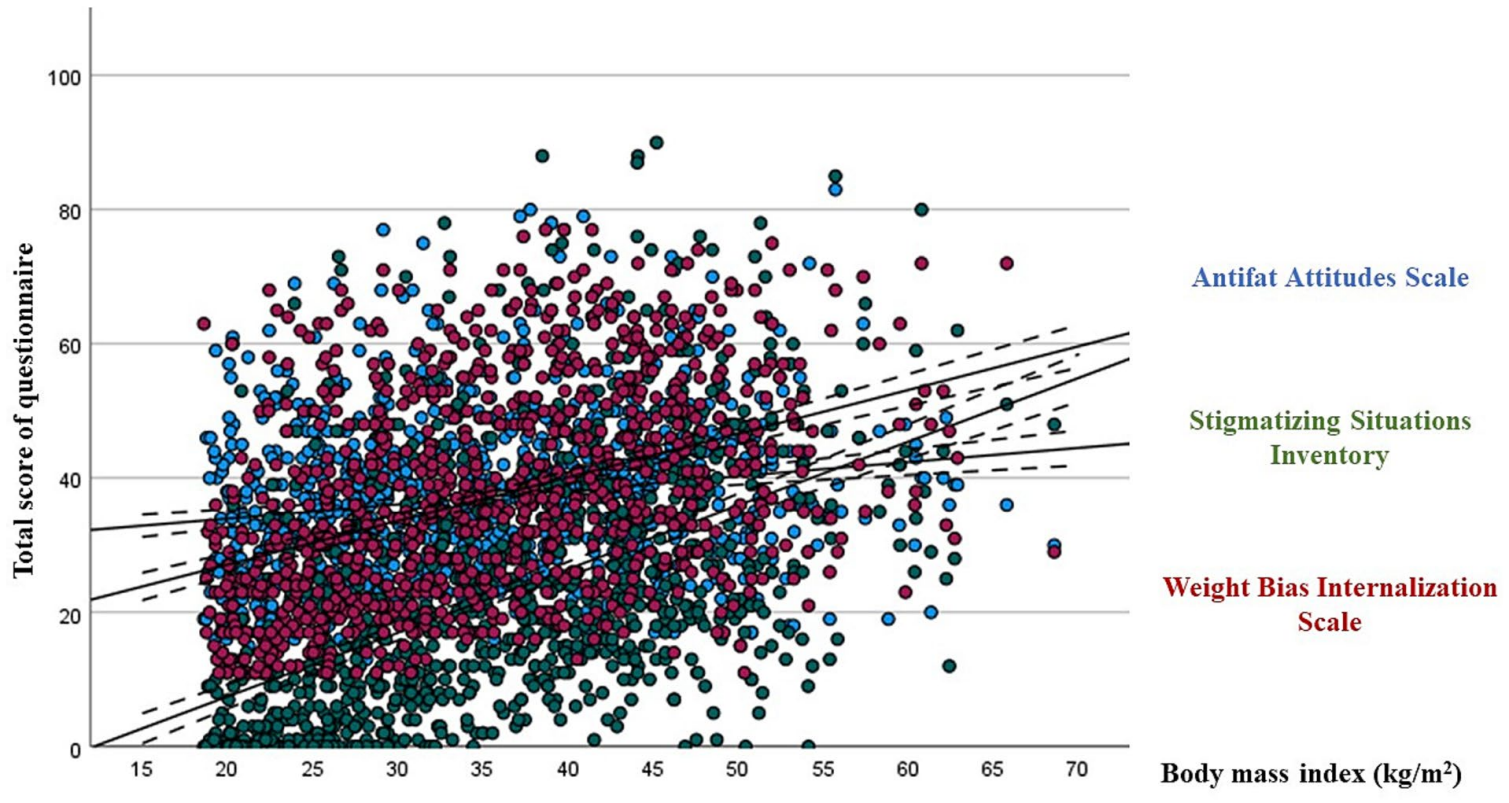
Bivariate correlations between body mass index and the three questionnaires.

In the bivariate analysis, BMI was significantly correlated with the total score of AFA (*r* = 0.171, *p* < 0.001), SSI (*r* = 0.522, *p* < 0.001) and WBIS (*r* = 0.417, *p* < 0.001) questionnaires (Figure 2). The multivariate analysis confirmed the differential role of BMI in all questionnaires (Table 3). In addition, the multivariate analysis also confirmed the differential role of age in relation to SSI and gender in relation to AFA and WBIS. However, neither employment status nor educational level were significant variables in any of the 3 questionnaires.

**Table 3:** A multivariable logistic regression model for total score of questionnaires in the whole population.

	*β*	Beta 95% CI	*P*
**Antifat attitudes scale (AFA)**			
Age	−0.057	–	0.213
Sex (M/F)	−3.074	−2.54 (−5.45 to −0.69)	0.011
BMI (kg/m^2^)	0.143	0.12 (0.02 to 0.26)	0.011
Employment status (Active/Unemployement)	0.918	–	0.081
Educational level (Basic/University)	−0.026	–	0.962
Constant		38.83 (29.97 to 47.68)	<0.001
*R*^2^ = 0.044			
**Stigmatizing situations inventory (SSI)**			
Age	−0.171	−0.12 (−0.30 to −0.41)	0.010
Sex (M/F)	3.077	–	0.081
BMI (kg/m^2^)	1.026	0.54 (0.85 to 1.19)	<0.001
Employment status (Active/Unemployement)	0.097	–	0.899
Educational level (Basic/University)	0.327	–	0.678
Constant		−12.49 (−25.32 to 0.33)	0.056
*R*^2^ = 0.272			
**Weight bias internalization scale (WBIS)**			
Age	–0.085	–	0.137
Sex (M/F)	3.096	0.09 (0.10 to 6.09)	0.043
BMI (kg/m^2^)	0.666	8.75 (0.52 to 0.82)	<0.001
Employment status (Active/Unemployement)	−0.390	–	0.556
Educational level (Basic/University)	−0.884	–	0.200
Constant		16.24 (5.03 to 27.44)	0.005
*R*^2^ = 0.192		–	

## Discussion

The OBESTIGMA study has measured prejudice against people with obesity and weight stigma in a large cohort of subjects with a wide range of BMIs in Spain. We have confirmed that individuals living with obesity encounter a heightened occurrence of experiences related to weight-related stigma. Furthermore, individuals with higher BMIs exhibit a pronounced belief that the prevailing biases about obesity in our society are applicable to their own circumstances. However, we observed that rather than observing a diminished level of prejudice toward individuals with obesity among those who are affected by the condition, our findings revealed the opposite trend: as the degree of obesity increased, so did the intensity of anti-obesity attitudes.

The main characteristics of the population encompassed in the OBESTIGMA study indicate that within participants with a BMI ≥30 kg/m^2^, both those possessing university degrees and those engaged in active employment experienced a decline. It is well known that mean BMI is higher among people with a lower lifetime socioeconomic status compared to those with a higher one, an association that is generally stronger among men than among women (Newton et al., 2017). Moreover, the prevalence of obesity has been associated with higher unemployment rates throughout Europe and in Spain (Diamantis et al., 2022). However, in our study, educational level and employment status do not seem to be independently related to the perception of discrimination and stigma due to obesity. While this outcome might appear unexpected, a recent systematic narrative review of the existing literature aimed at evaluating the potential influence of socioeconomic factors on weight-related stigma and discrimination yielded notably incongruent findings (Bernard et al., 2019). Among the eight studies conducted, six studies indicated a notable correlation between weight bias and education or income, while the remaining two studies presented opposing results, and an additional seven studies found no discernible association (Bernard et al., 2019).

Living with chronic illness is a complex, ever-evolving, cyclic, and multifaceted process (Ambrosio et al., 2015). In this way, obesity presents hurdles to psychosocial well-being and self-esteem, creating an ongoing and lifelong endeavor of embracing, coping, self-management, as well as striving for integration and adaptation in the face of this persistent and recurring condition (Christiansen et al., 2012; Ambrosio et al., 2015; Spahlholz et al., 2016). According to the AFA total score, patients with obesity had the strongest prejudices against being overweight, suggesting that much educational work remains to be done to increase acceptance of obesity among patients living with the disease. Among the three subscales assessed by the AFA questionnaire, both the apprehension about weight gain and the belief that obesity is influenced by individual control exhibited a direct correlation with the escalation in BMI. These results seem to contrast with a recent study by Macho et al. (2022) in which people with normal weight were those who showed more anti-obesity attitudes. This study recruited 1,248 participants (mean age 33.3 years, 66.5% of them with higher education) online from the Spanish population. This contrasts with the participants in the OBESTIGMA study, who were recruited within the clinical environment of Obesity Units. Therefore, our results highlight the need for further research on anti-fat attitudes using different samples to better understand the mechanism explaining weight discrimination and maintenance. As men showed more rejection towards people who were overweight or obese than women, this research will also require thinking about the gender perspective considering the attitudes, hopes and aspirations of each gender.

Stigmatizing weight experiences have a negative impact on people’s health and social behavior and have been associated with increased food intake in response to stress, increased exercise avoidance, and increased perceived stress (Pearl et al., 2021). These experiences could be grouped into direct (e.g., being abused when using public transport), environmental (e.g., not being able to fit into seats on planes), and indirect (e.g., people staring at the contents of their supermarket trolley) stigma (Link and Phelan, 2006). Interestingly, participants in the study of Lewis et al. (2011) described that more subtle forms of stigma had the most impact on their health and social wellbeing. As expected, in the OBESTIGMA study, negative weight experiences were more frequent among participants with higher BMI. However, a worrying observation was that weight-stigmatizing experiences were more frequent among younger participants, suggesting that modern life is a barrier to favoring the integration of people with obesity in our society. This result is in line with other two previous studies in nursing students, both in Turkey and in the United States, which show how moderate levels of fat phobia and negative attitudes towards people with obesity persist even among future health professionals (Darling and Atav, 2019; Usta et al., 2021). These findings only reinforce the idea that anti-fat attitudes and stereotyped perceptions need to be addressed during the early stages of the education within the general population (Elboim-Gabyzon et al., 2020).

The frequency and intensity of perceived weight discrimination limits all life projects (social, economic, educational, and psychological well-being) of people with obesity (Durso and Latner, 2008). In the WBIS questionnaire, we observed that individuals with obesity more frequently adopt negative beliefs and opinions about themselves due to external judgments from society. When people internalize weight stigma, negative emotional and physical health consequences develop, regardless of BMI (Durso and Latner, 2008). This leads to increased odds of 5–10% weight gain, poorer weight control behaviors, less food control, and lower eating self-efficacy (Ashmore et al., 2008; Puhl et al., 2021). In this context, various research converges on the counterintuitive conclusion that not recognizing oneself as being overweight or obese may be associated with more favorable physical and mental health outcomes than recognizing oneself as such (Robinson et al., 2020). A study conducted in the United States reported that approximately one in five adults in the general population and 52% of adults with obesity endorsed the highest levels of weight bias internalization. Those with the highest weight bias internalization tended to be white, had lower levels of education and income, were actively attempting weight loss, and had higher BMIs, greater self-perceived weight, and prior experiences of weight stigma, particularly teasing (Puhl et al., 2018). However, the score on the WBIS questionnaire does not always correlate with BMI in all studies, suggesting that the degree of internalization of weight bias does not necessarily depend on the degree of overweight of an individual (Durso and Latner, 2008). Our study’s multivariate analysis revealed a gender-specific role in relation to WBIS, with men obtaining higher scores. However, this finding was not corroborated in the study by Puhl et al. (2018) where among individuals with the highest weight bias internalization scores, 72.1% were women. Therefore, understanding the implications of internalized stigma can illuminate how individuals with obesity perceive themselves, assess their self-worth, and explore how societal attitudes might become internalized within their self-concept.

We did not find a significant reduction in the score of any of the 3 questionnaires in patients undergoing bariatric procedures compared with participants with obesity who did not undergo surgery. These results are in line with the study by Raves et al., in which the percentage change in BMI after bariatric surgery had no effect on any measure of weight-related stigma (Raves et al., 2016). It has been suggested that this lack of change is related to the fact that, even after radical weight loss, patients on average still fall into the obesity classification, which may not change the way society interacts with them (Bennett et al., 2022). In addition, patients who have chosen the surgical weight loss option run the risk of being re-characterized as “lazy” or “low effort” subjects (Fardouly and Vartanian, 2012). Third, memories of stigmatizing encounters persist in participants even after significant weight loss, and this may influence how they perceive their current encounters (Bennett et al., 2022). Indeed, this is an important line of research that needs to be continued to better understand the relationship between weight bias and bariatric surgery, particularly longitudinally.

Some limitations of our research should be noted. First, it is not clear whether a better definition of obesity, for example through percentage body fat, would change our results. Additionally, some of our participants self-reported their own weight and height, with the potential power to change our results (Lecube et al., 2020). Secondly, the cross-sectional design of our research hinders our ability to establish a causal relationship between attitudes towards obesity and the degree of excess weight. In addition, our study relies solely on self-reported data, which may be subject to recall bias or social desirability bias. Third, it’s important to note that our study population was drawn from different obesity units, potentially influencing the characteristics of participants and limiting their representativeness to the broader population (Ashmore et al., 2008). Lastly, unrecorded variables, including elements such as physical activity, dietary habits, coexisting medical conditions, medications, psychological factors, environmental influences, and the duration of obesity, could potentially introduce confounding effects in our results. Undoubtedly, future studies in a more representative population of the general population are necessary.

## Conclusion

The perception of obesity in Spain, encompassing both individuals with obesity and those with normal weight, is complex and divergent from our initial assumptions. Surprisingly, individuals with obesity exhibit a higher degree of aversion towards the disease compared to the population with normal weight. Concurrently, the experience of stigmatizing encounters shows a worrying increase among younger individuals. In light of these insights, mitigating negative attitudes, beliefs, and stigmatization related to obesity emerges as a pivotal element in the ongoing battle against this escalating public health concern. Lastly, it is noteworthy that bariatric surgery falls short of diminishing social stigma scores to levels equivalent to those observed in individuals with normal weight.

## Data availability statement

The raw data supporting the conclusions of this article will be made available by the authors, without undue reservation.

## Ethics statement

The studies involving humans were approved by the ethics committee of the Arnau de Vilanova University Hospital, approval number CEIC-2190. The studies were conducted in accordance with the local legislation and institutional requirements. The participants provided their written informed consent to participate in this study.

## Author contributions

ES and AC: conceptualization. AS and SG-M: data curation. ES and NV: formal analysis. LF and AM-S: investigation. FG and MS: methodology. JN and CM: software. AL: project administration and supervision. OD-T, GC, and SC: visualization. ES: writing – original draft. MC, CL-C, and AL: writing – review and editing. All authors contributed to the article and approved the submitted version.

## Funding

The Spanish Society for the Study of Obesity (Sociedad Española de Obesidad, SEEDO) supported this study.

## Conflict of interest

The authors declare that the research was conducted in the absence of any commercial or financial relationships that could be construed as a potential conflict of interest.

## Publisher’s note

All claims expressed in this article are solely those of the authors and do not necessarily represent those of their affiliated organizations, or those of the publisher, the editors and the reviewers. Any product that may be evaluated in this article, or claim that may be made by its manufacturer, is not guaranteed or endorsed by the publisher.
